# Fast Helical Tomotherapy in a head and neck cancer planning study: is time priceless?

**DOI:** 10.1186/s13014-015-0556-8

**Published:** 2015-12-23

**Authors:** Dirk Van Gestel, Geert De Kerf, Kristien Wouters, Wouter Crijns, Jan B. Vermorken, Vincent Gregoire, Dirk Verellen

**Affiliations:** University Radiotherapy department Antwerp (URA), Antwerp, Belgium; Department of Radiotherapy, Institut Jules Bordet, Université Libre de Bruxelles, Rue Héger Bordet 1, 1000 Brussels, Belgium; Iridium Cancer Network GZA, Wilrijk, Belgium; Scientific Coordination and Biostatistics, Antwerp University Hospital, Edegem, Belgium; Department of Radiation Oncology, Leuvens Kankerinstituut, Leuven, Belgium; Faculty of Medicine and Health Sciences, University of Antwerp, Antwerp, Belgium; Department of Medical Oncology, Antwerp University Hospital, Edegem, Belgium; Radiation Oncology Department & Centre for Molecular Imaging and Experimental Radiotherapy, St-Luc University Hospital, Brussels, Belgium; Radiotherapy UZ Brussel, Faculty of Medicine and Pharmacy Vrije Universiteit Brussel, Brussels, Belgium

**Keywords:** Head-and-Neck Cancer, Helical tomotherapy, Treatment time, Treatment quality

## Abstract

**Background:**

The last few years, in radiotherapy there has been a growing focus on speed of treatment delivery (largely driven by economical and commercial interests). This study investigates the influence of treatment time on plan quality for helical tomotherapy (HT), using delivery times with Volumetric Modulated Arc Therapy (VMAT; Rapid Arc [RA]) as reference.

**Methods:**

In a previous study, double arc RA (Eclipse) and standard HT plans (TomoHD™) were created for five oropharyngeal cancer patients and reported according to ICRU 83 guidelines. By modifying the beam width from 2.5 to 5.0 cm, elevating the pitch and lowering the modulation factor, “TomoFast” (TF) plans were generated with treatment times equal to RA plans. To quantify the impact of TF’s craniocaudal gradient, similar plans were generated on TomoEdge^TM^ (TomoEdgeFast;TEF). The homogeneity index (HI), conformity index (CI), mean dose, D_near-max_ (D2) and D_near-min_ (D98) of the PTVs were analyzed as well as the mean dose, specific critical doses and volumes of 26 organs at risk (OARs). Data were analyzed using repeated measures ANOVA.

**Results:**

With a mean treatment time of 3.05 min (RA), 2.89 min (TF) and 2.95 min (TEF), PTV_therapeutic_ coverage was more homogeneous with TF (HI.07;SE.01) and TEF (HI.08;SE.01) compared to RA (HI.10;SE.01), while PTV_prophylactic_ was most homogeneous with RA. Mean doses to parotid glands were comparable for RA, TF, TEF: 25.62, 25.34, 23.09 Gy for contralateral and 32.02, 31.96, 30.01 Gy for ipsilateral glands, respectively. OARs’ mean doses varied between different approaches not favoring a particular technique. TF’s higher dose to OARs at the cranial-caudal edges of the PTVs and its higher integral dose, both due to the extended cranial-caudal gradient, seems to be solved by the new TomoEdge™ software. However, all these faster techniques lose part of standard TomoHD’s OAR sparing capacity

**Conclusion:**

It is possible to treat oropharyngeal cancer patients using HT (TF/TEF) within time-frames observed for RA maintaining comparable target coverage and sparing of OARs. This study indicates that treatment time is not technology specific, rather an operator’s decision on balancing efficiency and quality.

## Background

The last few years rotational Intensity-Modulated Radiotherapy (IMRT) techniques have become more popular. They are gradually replacing the older static beam IMRT techniques mainly based on faster fraction delivery with at least equal, and often better, sparing of the organs at risk (OARs) [1]. In a previous planning study on oropharyngeal cancer we showed the potential of helical tomotherapy (HT; Accuray, Sunnyvale, CA) compared to the ‘cone beam’ or ‘volumetric’ rotational IMRT techniques such as RapidArc (RA; Varian, Palo Alto, CA) planned with Eclipse (Varian, Palo Alto, CA) and Elekta VMAT (Elekta, Stockholm, Sweden) planned with Pinnacle’s SmartArc (Philips, Eindhoven, The Netherlands) in terms of sparing of the OARs of the neck [2]. However, treatment time with standard HT was nearly twice that of RapidArc planned by Eclipse (3.05 vs 5.94 min). Next to a negative impact on patient comfort, treatment accuracy (more intrafractional motion, i.e. motion during one fraction) and department economics, this longer treatment time potentially may also have a negative effect on tumor control. Of interest in this respect is a nasopharyngeal carcinoma cell culture experiment by Zheng et al. showing longer fraction delivery times to give less tumor cell kill, probably due to sub-lethal damage repair during the radiotherapy session [3]. However, that study compared delivery times of 15 and 50 min and it is not sure whether their results are also applicable on delivery times between 3 and 6 min. On the other hand, although the total treatment time is clearly longer with HT than with RA, the treatment time of a single point (tumor cell) within the target volume is much shorter and can be as low as 42 s at maximum gantry speed (12 s per gantry rotation), for a standard pitch of .287 [4]. This faster local dose delivery might result in less sub-lethal damage repair and therefore more lethal damage during a single treatment session. Shaikh et al. estimated an increase in the tumor control probability (TCP) of 2–3 % with HT compared with the faster 3D conformal RT due to the lower dose rate of the latter, and an additional 2–3 % compared with static-beam IMRT [5].

An additional argument against the longer treatment times is based on economic and commercial considerations [6, 7].

For all these reasons, in this study, it was decided to speed up the HT system to treatment times comparable to those obtained with RA (RA was used as benchmark as it can be considered being more mainstream) and to quantify the consequences on the quality of the treatment plan. This acceleration is possible by varying 3 separate parameters in the HT planning’s system: the beam width, the pitch and the modulation factor. The beam width is the cranio-caudal aperture of HTs fan beam multileaf collimator (MLC), which we would call the length in a classical cone beam collimator. One can choose between 1, 2.5 and 5 cm. With increasing beam width also the craniocaudal gradient/penumbra increases due to the typical characteristics of the fan beam MLC of the actual TomoHD™ and older systems [8, 9]. The recently released TomoEdge™ Dynamic Jaws of the TomoHDA™ system, referred to by Sterzing et al. and Rong et al. as the ‘running-start-stop delivery’, aims to resolve this issue by gradually adapting the beam width at the craniocaudal outer ends of the target volume [9–11]. The pitch is the distance covered by the treatment couch relative to the beam width, during one 360° rotation of the accelerator around the patient. As such, a pitch of 0.5 indicates that the treatment couch advances 50 % of the beam width during each rotation, which means that each point within the tumor/patient can be irradiated/optimized during 2 full rotations. Finally, the modulation factor is the maximally accepted level of modulation. A larger beam width, a larger pitch or a smaller modulation factor, can each speed up the HT treatment.

In this planning study for head and neck cancer patients we plan to equal the treatment time parameter of HT with the one obtained by RA. Both will be compared with the standard HT plans in order to better understand the dosimetric consequences of a faster treatment. Finally, the impact of the new TomoEdge™ system will be evaluated under fast conditions.

## Methods

Only a brief summary is provided on patient characteristics and treatment planning as these are based on a previous planning study and the reader is kindly referred to that publication for more details [2].

### Patient characteristics

For five patients with loco-regionally advanced oropharyngeal cancer a contrast-enhanced reference CT scan with 3 mm slice thickness was made in the treatment position with a custom made immobilization mask. Patient characteristics are mentioned in Table 1. Primary tumor volume and bilateral elective lymph node regions and OARs were delineated.

**Table 1:** Patient characteristics

Patient	Location	ICD-O 10	Classification TNM/AJCC VI	Stage	LN+ level	PTV_therapeutic_ volume (cm^3^)	PTV_total_ volume (cm^3^)
1	Base of tongue R	C01	T1N2aM0	IV A	2 & 3 R	104	483
2	Tonsil R	C09	T2N2cM0	IV A	2 bilat & 3 R	233	610
3	Tonsil L	C09	T3N2cM0	IV A	bilat 1,2,3,4	380	955
4	Base of tongue R	C01	T3N2cM0	IV A	1b R & 2 bilat	422	831
5	Tonsil L	C09	T2N1M0	III	2 L	146	563
average						257	688

*Abbreviations: Bilat* bilateral, *LN+* positive lymph node, *R* right, *L* left, *PTV*_*total*_ PTV_therapeutic_ and PTV_prophylactic_ together; PTV volumes reported in this table are calculated by the Pinnacle treatment planning system

### Treatment planning

#### Volumes

The gross tumor volume (GTV), the clinical target volume (CTV) and the nearby organs at risk (OARs) were delineated on the same software platform for all treatment plans (Pinnacle 8.0 m). The CTV_69Gy_ (i.e. the CTVtherapeutic) was defined as the GTV + 1 cm (both for the primary tumor and for the regional lymph node metastasis) respecting the anatomical limitations of spread by bone, cartilages, ligaments, muscles and air. The rest of the CTV of both the primary tumor (tissue nearby at risk of direct spread) and the bilateral elective lymph node areas (delineated according to Gregoire et al. [12]) were united in the CTV_56Gy_ (i.e. the CTV_prophylactic_). The planning target volumes (PTVs) 69 and 56 Gy were defined as the respective CTVs plus a 3 mm margin with exclusion of the skin. This skin, defined as a 3-mm thick layer under the surface, was excluded from the PTV.^1^ The PTV_69Gy_ was created as a separated volume, i.e. was not included in the PTV_56Gy_, to bypass HT’s overlap priority system in which a single pixel can only represent one target volume.

The contoured OARs are listed in Table 2. The shoulder was delineated as the humeral head, including the glenohumeral joint, up to the acromioclavicular joint. The top of the lung is defined as the cranial part of the lung above the aortic arc. OARs lying (almost) completely in the PTV were not contoured. A planning risk volume (PRV) of 3 mm was created around the spinal cord and around the brainstem.

**Table 2:** Dose-volume constraints for PTVs and organs at risk

Target/Organ at risk	Median absorbed dose or D50 %	Mean absorbed dose	ALARA	D_near-min_ or D98 %	D_near-max_ or D2 %
PTV_56Gy_	56 Gy		V59.9 Gy	≥95 % of planned absorbed dose	
PTV_69Gy_	69.12 Gy			≥95 % of planned absorbed dose	≤107 % of planned absorbed dose
PRV Spinal cord			D2		≤50 Gy
PRV Brainstem	≤55 Gy		D2		≤59 Gy
Parotid gland contralateral		≤23 Gy	Mean D, V27		
Parotid gland ipsilateral		≤27 Gy	Mean D, V27		
Submandibular gland		≤39 Gy	Mean D		
Oral mucosa		≤27 Gy	Mean D, V27		
Mandible			V60		
Soft palate		≤27 Gy	Mean D, V27		
Constrictor muscles		≤55 Gy	Mean D, V20		
Cricopharyngeal muscle		≤55 Gy	Mean D, V20		
Base of tongue		≤55 Gy	Mean D, V20		
Larynx		≤40 Gy	Mean D, V40		
Esophagus superior		≤35 Gy	Mean D, V35		
Top of lung			V20		
Inner ear			Mean D, V45		

*Abbreviations: PTV* planning target volume, *PRV* planning risk volume, *D* dose, *V* volume, *ALARA* as low as reasonable achievable

#### Planning techniques

For each patient a treatment plan was made in the institution where a specific form of IMRT was in use. The Tomotherapy plans were all made in the same institution by the same physicist and finalized by the same radiation oncologist. Techniques 1 and 2 used in this study are identical to those used in a previous study, and serve as comparators for techniques 3 and 4 [2].

1. A Helical Tomotherapy plan for a TomoHD™ system was planned on the Tomotherapy planning software version HD1.0 with a maximum of three dose volume histogram (DVH) control points per volume. A field width of 2.5 cm, a maximum modulation factor of 2.8 and a pitch of 0.287 (to avoid the thread effect [13]) were used. The dose distribution for each beamlet was calculated with a convolution/superposition algorithm. The optimization process used the least mean square optimization method to optimize the objective function.
2. A RapidArc treatment was planned for a Varian CLINAC 2100 C/D upgraded with on board imaging (OBI) and RapidArc. The plans were optimized using the Progressive Resolution Optimizer (PRO) 8.6.15 and calculated with Anisotropic Analytical Algorithm (AAA 8.6.15). Each plan consisted of two 6 MV 360° arcs, one clockwise (CW) and one counter clockwise (CCW) of 177 control points each. To avoid “tongue-and-groove” effects and to improve target coverage and OAR protection, collimator angles were set to 10° (CCW) and 80° (CW) [14]*.* The collimator used was a 120 MLC for a 40 by 40 cm field size (5 mm leaf width for the central 20 cm, 10 mm leaf width for the outer 20 cm).
3. With the same Tomotherapy planning software version HD1.0 a “Tomo Fast” (TF) plan was created. By modifying the beam width from 2.5 to 5.0 cm, elevating the pitch to 0.43 and lowering the modulation factor from an initial 2.8 down to the moment the required treatment time was reached, plans with treatment times close to those of the RA plans could be generated.
4. With the same technique as the TF plans, plans were made for the recently released TomoEdge™ Dynamic Jaws system of the TomoHDA™ series (TEF) [9–11]. With this new hardware it is possible to gradually open the fan beam jaws at the craniocaudal outer ends of target volume, avoiding the typical larger craniocaudal penumbra of HT. The dose distributions are calculated with the VoLO™ algorithm using a convolution/superposition algorithm.

#### Prescription and constraints

A simultaneous integrated boost technique was planned in order to deliver in 32 fractions a dose of 69.12 Gy (2.16 Gy / fraction) to the PTV_69Gy_ and a dose of 56 Gy (1.75 Gy / fraction) to the PTV_56Gy_, respecting the prescription guidelines of the International Commission on Radiation Units and Measurements (ICRU) report 83 [15].

The Near-Max dose (D_near-max_ or D_2%_) to the PRV of the spinal cord was limited to 50 Gy and a maximum of 59 Gy with 50 % of the volume (D_50%_) under 55 Gy was tolerated to the PRV of the brainstem. The dose to all OARs had to be kept as low as possible respecting the prescription to the PTVs. An overview of the dose-volume constraints for the PTVs and the different OARs can be found in Table 2.

### Data analysis and statistics

#### Reporting

For the PTVs the homogeneity index (HI, (D_2%_-D_98%_)/D_50%_), the conformity index (CI, V_95%_D_prescribed(body)_/V_95%_D_prescribed(PTV)_), the mean dose, the D_near-min_ (D_98%_) and the D_near-max_ (D_2%_) were analyzed. The V_59.9Gy_ of the PTV_56Gy_ (=107 % of the prescribed dose) was calculated as an indication for the steepness of the dose gradient towards the PTV_69Gy_. For 30 OARs the mean dose and specific critical doses and volumes were analyzed. Organs overlapping with the PTV in three patients or more were excluded from this study. Finally the beam-on time, treatment time and the number of monitor units were compared.

#### Analysis and statistics

Differences in the studied parameters between treatment planning systems were analyzed using the general linear model in the form of a repeated measures analysis of variance (ANOVA). By using a heterogeneous covariance structure in the repeated measures model, we allowed the variance to differ across systems. All included variables were checked for normality. The *p*-values of group comparisons were adjusted for multiple testing using the false discovery rate (FDR) correction. All hypotheses were tested non-directionally with a *p*-value of less than 0.05 considered to be significant. All analyses were performed using SAS 9.2 (SAS Institute Inc., Cary, NC).

## Results

For HT and RA the results of our previous planning study were used [2]. The modulation factor for TF and TEF was lowered from 2.8 to a mean of 2.30 (range 2.0–2.6) and 2.36 (range 2.0–2.8), respectively.

### PTVs

**Fig. 1:**
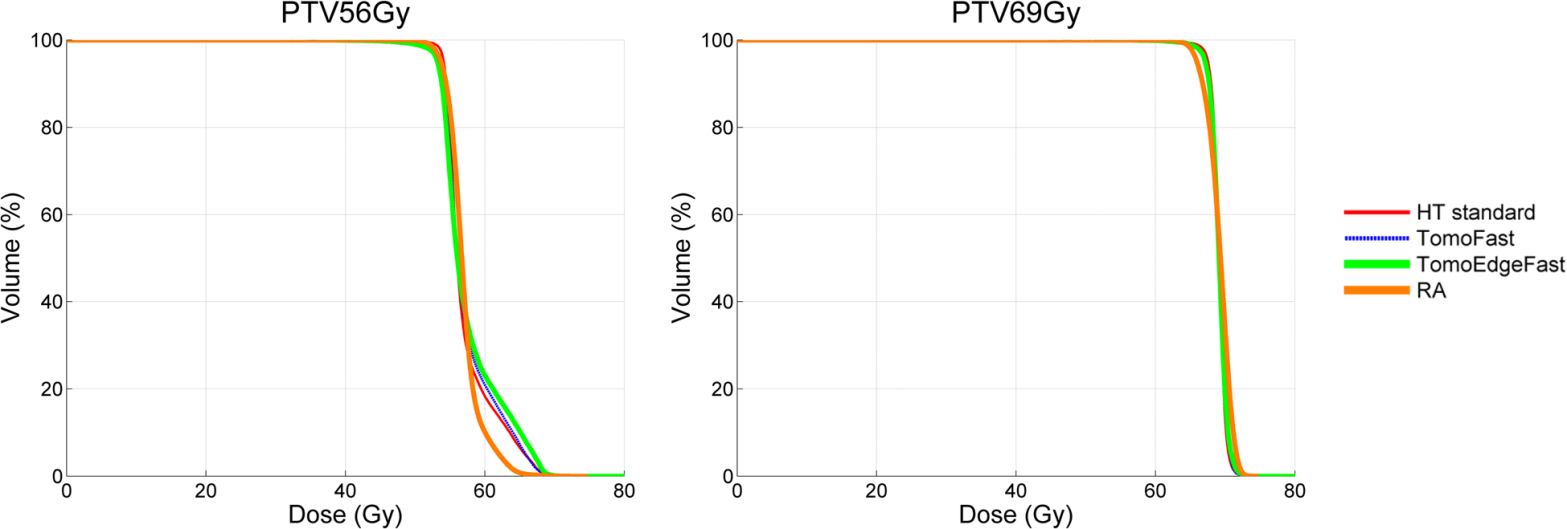
Composite dose volume histograms of mean PTV_69Gy_ and mean PTV_56Gy,_ with averaged data from the five plans

An overview of the mean results and corresponding *p*-values for PTV_69Gy_ and PTV_56Gy_ is given in Table 3, the corresponding dose volume histograms can be found in Fig. 1.

**Table 3:**
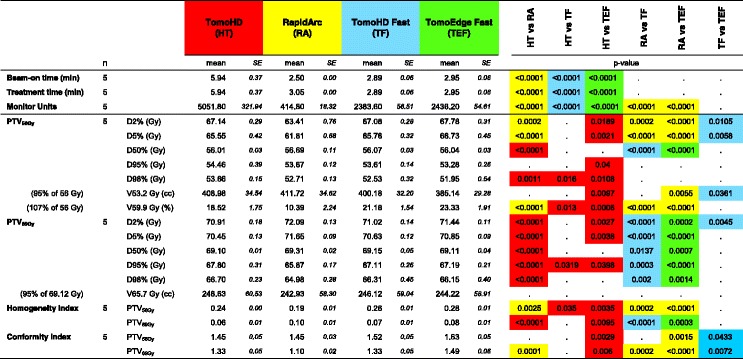
Mean values of PTVs for the different planning techniques

*Abbreviations: PTV* planning target volume

The left side of the table gives the mean values of the PTVs. the right part of the table indicates the statistically significant differences (Repeated Measures ANOVA) between the five techniques. each with its own colour highlighting the technique doing best in the comparison: red = Helical TomoHD; yellow = RapidArc VMAT; blue = TomoHD Fast; green = TomoEdge Fast. For HT and RA the results of our previous planning study were used [2]

**PTV**_**69Gy**_**:** D_2%_ and D_50%_ ICRU 83 guidelines were well respected by all techniques, the D98% guideline was respected by HT, TF and TEF. This resulted in a statistically significant lower homogeneity index (mean HI .06, .07 and .08 for HT, TF and TEF, respectively) compared to RA (mean HI .10). The mean Conformity Index was best for RA (1.10), remained the same for TF compared to HT (1.33), but was clearly worse for TEF (1.49).

**PTV**_**56Gy**_**:** the D_50%_ guideline was less well respected by RA, D_98%_ was only respected by HT and D_2%_ by none. A statistically significant lowest homogeneity index was obtained by RA (.19), and HT (.24) did better than TF and TEF (.26 and .28, respectively). The Conformity index PTV_56Gy_ was worst for TEF.

### OARs

For 26 organs at risk, an extensive list of mean values and organ specific critical doses and volumes is given in Table 4. Mean doses to the parotid glands, for example, were comparable for RA, TF and TEF: 25.62 Gy, 25.34 Gy and 23.09 for the contralateral and 32.02 Gy, 31.96 Gy and 30.01 for the ipsilateral gland, respectively. When comparing the fast techniques, spinal cord, cricopharyngeal muscle and cranial part of the esophagus received a lower mean dose when planned with TF or TEF, the supraglottic larynx when planned with RA.

**Table 4:**
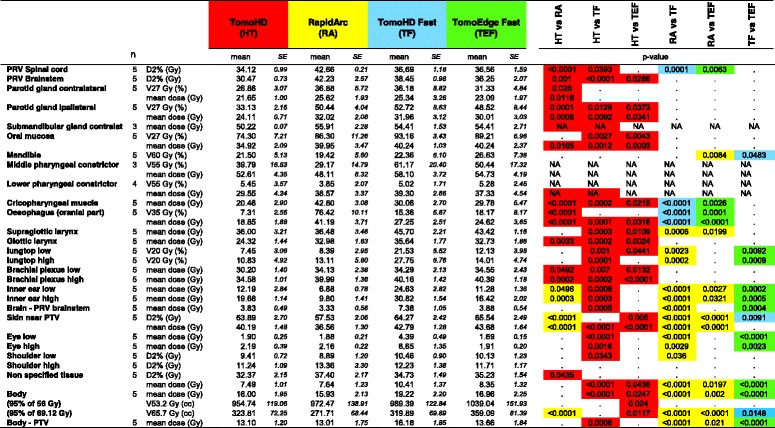
Critical doses and volumes of the different organs at risk for the different planning techniques

*Abbreviations: PRV* planning risk volume, *PTV* planning target volume, *SE* standard error; high and low: was used in symmetrical organs where ‘ipsi- and contralateral’ was not appropriate; *NA* not analysed because of the low number of parameters

The left side of the table gives the mean values of the OARs. the right part of the table indicates the statistically significant differences (Repeated Measures ANOVA) between the five techniques. each with its own colour highlighting the technique doing best in the comparison: red = Helical TomoHD; yellow = RapidArc VMAT; blue = TomoHD Fast; green = TomoEdge Fast. For HT and RA the results of our previous planning study were used [2]

**Fig. 2:**
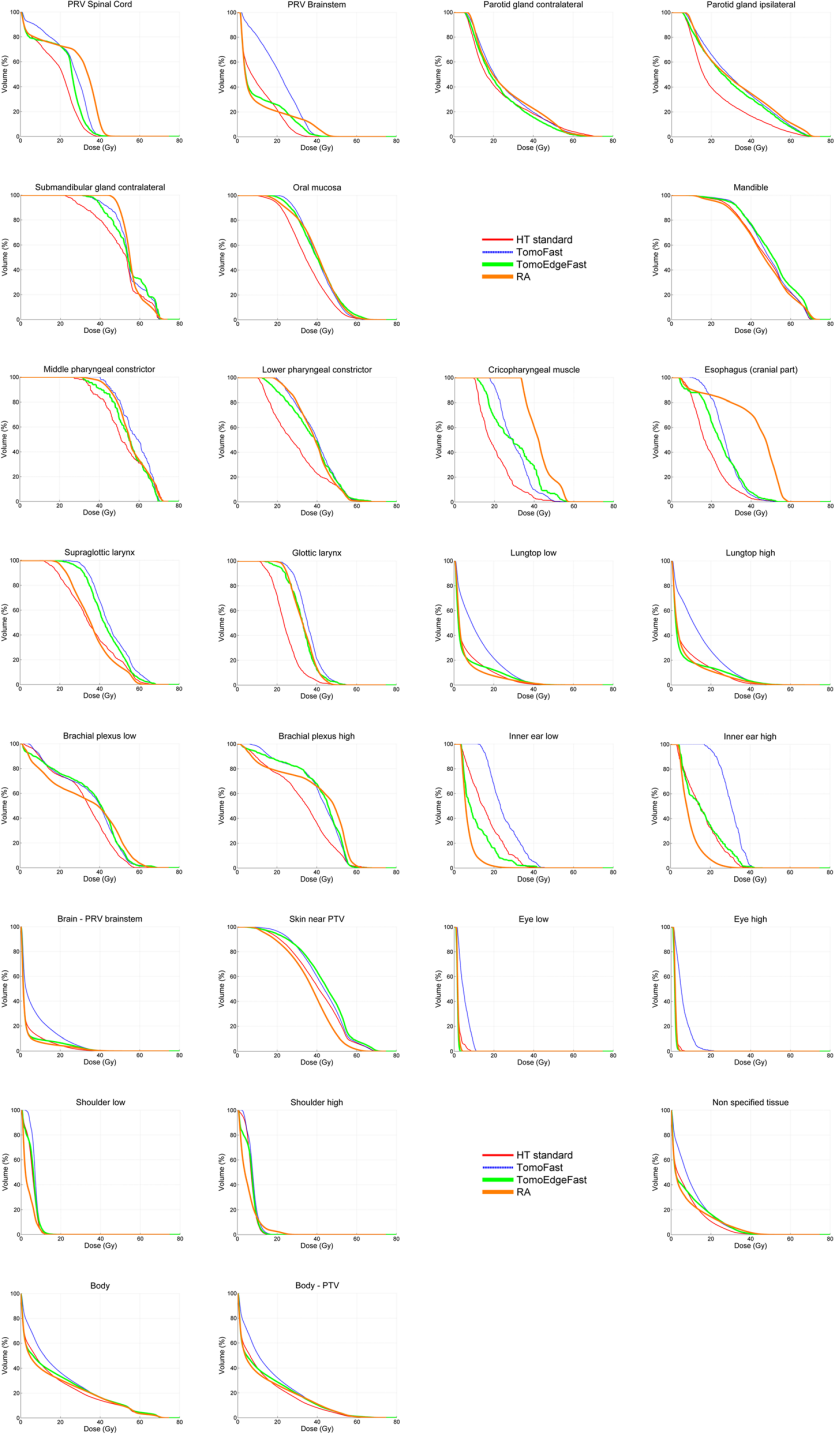
Composite dose volume histograms of mean values of organs at risk and body, with averaged data from the five plans

At the same time, these fast techniques lose the dosimetric advantage of HT. The composite dose volume histograms pooled from the five plans of the OARs and the total body can be found in Fig. 2.

#### Monitor units, beam-on time and treatment time (see Table 3)

The mean number of *monitor units* was statistically lower for RA (415) compared to TF (2384) and TEF (2436), both requiring half the MU of HT (5052). Mean *beam-on time* was 2.5 min for RA, 2.9 min for TF and TEF, and 5.9 min for HT. *Treatment times* (excluding patient set-up) were 3.05 min for RA and remained the same as the beam on time for the 3 helical tomotherapy techniques.

## Discussion

Typically, all rotational IMRT systems are performing quite well with the faster techniques (RA, TF and TEF) keeping the target volume coverage acceptable, but compromising the sparing of organs at risk.

Globally, the target volume coverage is the best with standard HT, yet acceptable results are obtained with the faster techniques. Especially the difference with TF is minimal in this study. TF and TEF score better than RA for the PTV_69Gy_ but worse for the PTV_56Gy._ This can be explained by the placing of the dose gradient between both dose levels which, in this study, is placed in the elective/prophylactic dose zone in the Tomotherapy techniques and in the high dose zone in RA. It remains unclear whether the place of the dose gradient is inherent to the planning system or whether it is planner/institution driven. The clinical relevance of the observed differences in PTV coverage can be argued. The moderate CI for standard HT, especially in comparison with RA, is explained by the high D98 % which pushes a bigger part of the D95 % outside the PTV. This implies that we might have made these plans to homogeneous, maybe at the expense of an even better sparing of the OARs.

For most of the OARs, TF and TEF lose the standard HT’s dosimetric advantage over RA and thus also the probable clinical benefit in parotid gland sparing, swallowing function preservation and easier re-irradiation as discussed in our previous article [2]. Differences in OAR sparing between the three faster techniques are very small with almost similar mean dose to both parotids and the oral mucosa. Furthermore, some OARs are doing better with TF/TEF (Spinal cord, cricopharyngeal muscle and cranial part of the esophagus), while others (e.g. supraglottic larynx) are doing better when planned with RA. For the OARs at the cranio-caudal edges of the PTV (e.g. eyes, ears, lung tops, brain and shoulders), TF is doing worse than RA due to the craniocaudal penumbra caused by the open/close fan beam. With exception of the ears, this problem is not present in the TEF planning, as the TomoEdge™ Dynamic Jaws of the TomoHDA^TM^ series makes it possible to gradually adapt the beam width at the craniocaudal edges of the target volume, reducing the cranio-caudal penumbra [9–11]. This edge effect also translates into a 20 % higher integral dose for TF compared to standard HT, while TEF recuperates a large part of this disadvantage (only 6 % higher integral dose). This corresponds to the findings of Sterzing et al. in a preclinical planningstudy on 10 nasopharyngeal cancer cases observing a 12.5 % increase and a 5.7 % decrease of the integral dose, respectively [9]. However, for TEF the same group recently reported a 3.1 % increase in integral dose in a mixed tumor population in a clinical setting [11]. We found TEF to have an inferior CI compared to TF, which is rather surprising. A similar observation has been made in the studies of Sterzing et al. and of Rong et al. [9, 10]. This can possibly be explained by the fact that the VoLO algorithm of the TomoEdge™ system, in order to speed up calculation time, skips the time consuming beamlet calculation which makes use of the Collapsed Cone Convolution Superposition (CCCS) approach. It immediately starts with the optimization process using a new Fluence Convolution Broad Beam (FCBB) dose calculation in combination with a full scatter dose calculation every ten iterations. This different approach can probably result in minor differences in the calculated dose distribution [16].

However, the study of Sterzing et al. was performed on a prototype which has afterwards been recognized not to be suitable for clinical use. The only other studies so far that report on the clinical released TomoEdge system are the studies by Rong and colleagues and the recent study of Katayama et al. [10, 11]. The first reported on 20 patient cases of which four head and neck cancer (HNC) cases; the latter on 45 patients of which 16 HNC cases. However, most of these HNC cases were not very challenging, originated from different head and neck sites, had different dose prescriptions and only very few OARs were reported on. The main focus of both studies was a safe faster treatment delivery by the TomoEdge system but they failed to uniformly quantify the dosimetrical impact on the OARs. In our study, on the other hand, we found a clear dosimetrical impact of the faster delivery in comparison to the standard HT plan.

A faster treatment delivery might be beneficial for intrafractional motion and for the patient who might stay for a shorter period of time under the immobilization mask. However, the importance of reducing treatment times from 6 to about 3 min can be questioned given the overall in-room time that is much longer due to the daily repositioning of the patient and the image guidance. Nevertheless, with time slots of 20 min, a gain of 3 min per patient (=15 %) with the faster techniques results in an extra four patients that can benefit of an IMRT technique during the same 8 h working day compared to standard HT. This might not be an issue in academic centers with enough machine time, but it might be an issue in smaller hospitals.

Moreover, we already pointed out that a faster treatment delivery possibly is beneficial by avoiding cell kill repair and how HT therefore might result in an increase in the tumor control probability [3, 5]. As standard HT is already treating an individual point/voxel much faster than other techniques (42 s at maximum gantry speed for a pitch of .287), TF and TEF further decrease this time to minimally 28 s for a pitch of .43 used with the 5 cm beam width. Hence, a further increase in the tumor control probability might be expected. However, we realize that this hypothesis is merely speculative.

We also realize that the statistical power of a sample size of only five patients (each of them planned with the four planning systems) has its limitations. Since normality of the results of such a small sample cannot be proven, non-parametric tests would be the first choice. However, with Wilcoxon signed rank tests it is theoretically impossible to get a p-value of less than .05 in such a small group. Visual inspection of the data by means of boxplots and qq-plots showed large differences between the different techniques and no major deviations from normality. Therefore repeated measures ANOVA models were performed. When differences are sufficiently large and the variability within each system is small, as is the case in the present study, repeated measures ANOVA has the power to pick up these differences. However, the authors realize that smaller differences may be missed as non-significant results may be due to the low number of patients, and thus not necessarily implement that the different systems are equal for these parameters. Another possible bias encountered in the present study is the fact that the RA and HT plans have been planned by different institutes and thus different planners. This is beneficial in that each technique has been planned by the most experienced planner, but it might introduce uncertainty whether the observed differences are not due to the planner rather than being technique specific. This is true for the comparison between RA and HT but is invalid for the comparison among the three HT techniques as they all have been planned in the same way by the same planners. As the impact of treatment time on the HT plan quality was the main goal of our study and as the RA plans were only referred to as a more mainstream benchmark, we are quite confident that differences in planner introduced only minor influences to the endpoints of our study.

Finally, this study was limited to two situations, a standard plan and a fast plan, but an infinite number of combinations of pitch, width and modulation factor, each with its specific characteristics can be explored. In order to guide the planner’s choice for the case specific best parameters, a HT specific Pareto front has been developed which has been subject of another publication [17].

## Conclusion

HT can be speeded up to about half the original treatment time, obviously at the cost of less sparing of the OARs. Lately, the focus in radiotherapy has been shifted towards speed of treatment delivery (largely driven by economical and commercial interests). With this paper we hope to show that speed is an artificial argument inferior to quality. The choice is not technology dependent, rather a decision made by the user to find the correct balance.

## Footnotes

1 This has been described in a previous study where it has been done in order to avoid 1) optimization problems with the static beam IMRT systems due to their physical inability to create proper dose in the build-up zone, in this case the skin; and 2) overdose to the skin created by the rotational IMRT systems [2].
